# Roles of VMP1 in Autophagy and ER–Membrane Contact: Potential Implications in Neurodegenerative Disorders

**DOI:** 10.3389/fnmol.2020.00042

**Published:** 2020-03-31

**Authors:** Panpan Wang, Daqing Kou, Weidong Le

**Affiliations:** ^1^Liaoning Provincial Center for Clinical Research on Neurological Diseases, The First Affiliated Hospital, Dalian Medical University, Dalian, China; ^2^Liaoning Provincial Key Laboratory for Research on the Pathogenic Mechanisms of Neurological Diseases, The First Affiliated Hospital, Dalian Medical University, Dalian, China; ^3^Department of Clinical Laboratory, The First Affiliated Hospital, Dalian Medical University, Dalian, China

**Keywords:** endoplasmic reticulum, autophagy, membrane contact sites, neurodegenerative disorders, VMP1

## Abstract

Cellular communication processes are highly dynamic and mediated, at least in part, by contacts between various membrane structures. The endoplasmic reticulum (ER), the major biosynthetic organelle of the cell, establishes an extensive network with other membrane structures to regulate the transport of intracellular molecules. Vacuole membrane protein 1 (VMP1), an ER-localized metazoan-specific protein, plays important roles in the formation of autophagosomes and communication between the ER and other organelles, including mitochondria, autophagosome precursor membranes, Golgi, lipid droplets, and endosomes. Increasing evidence has indicated that autophagy and ER–membrane communication at membrane contact sites are closely related to neurodegenerative disorders, such as Parkinson’s disease, Alzheimer’s disease, and amyotrophic lateral sclerosis. In this review, we summarize the roles of VMP1 in autophagy and ER–membrane contacts and discuss their potential implications in neurodegenerative disorders.

## Introduction

Vacuole membrane protein 1 (VMP1), a pancreatitis-associated protein (Dusetti et al., 2002; Vaccaro et al., 2003), has attracted attention owing to its modulatory effects on membrane trafficking and autophagy. VMP1 is an endoplasmic reticulum (ER)-resident, multi-spanning membrane protein and a candidate marker for autophagosome formation (Ropolo et al., 2007; Vaccaro et al., 2008). The expression of VMP1 can be induced by starvation and rapamycin treatment, and its overexpression triggers conversion of microtubule-associated protein 1 light chain 3 (LC3)-I to LC3-II and autophagosome formation in mammalian cells (Ropolo et al., 2007). Recently, several studies have shown that VMP1 is enriched in ER microdomains that are closely associated with diverse organelles. Tabara and Escalante (2016) were the first to demonstrate the highly dynamic nature of VMP1 puncta in concert with lipid droplets (LDs), mitochondria, and endosomes. Subsequently, Zhao et al. (2017) further confirmed that VMP1 modulates the contacts between the ER and isolation membranes (IMs), also between the ER and other organelles, including mitochondria, LDs, and endosomes.

**FIGURE 1:**
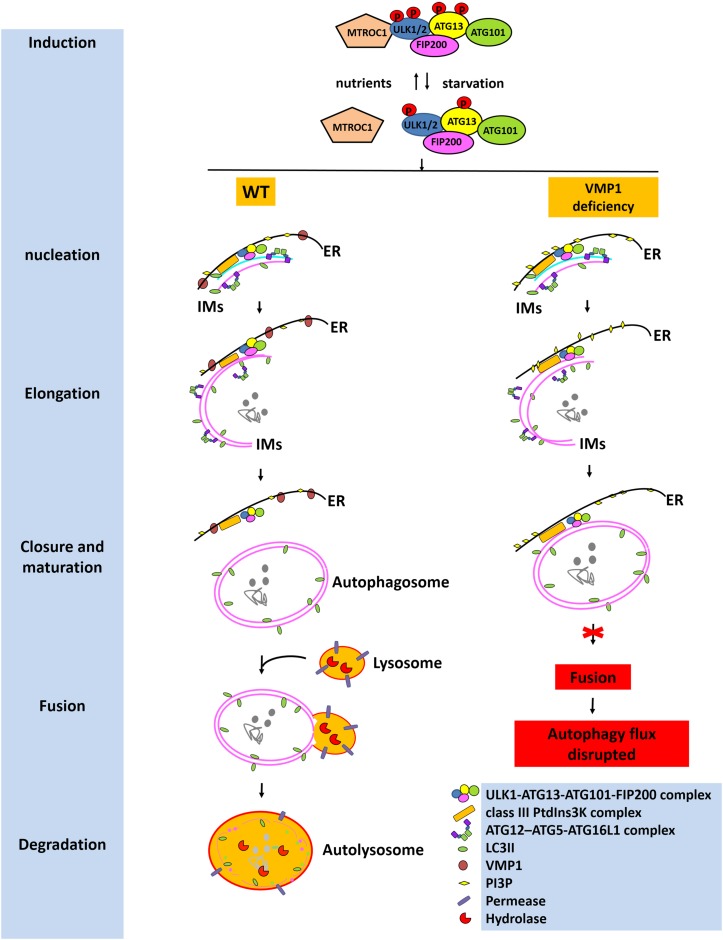
Roles of VMP1 in autophagy. Under starvation, MTORC1 dissociates from the ULK1/2 complex consisting of ULK1/2-ATG13-ATG101-FIP200; then ATG13 and ULK1/2 are partially dephosphorylated by as yet unidentified phosphatases, allowing the induction of macroautophagy. Nucleation and elongation of the IMs are aided by the class III PtdIns3K complex and ATG12-ATG5-ATG16L1 complex. Eventually, the expanding membrane closes around its cargo and separates from the ER to form a mature autophagosome. LC3-II is cleaved from the outer membrane of the autophagosome. The outer membrane of the autophagosome will then fuse with the lysosomal membrane to form an autolysosome. The contents of the autolysosome are then degraded and exported back into the cytoplasm for recycling. However, VMP1 depletion will lead to tight contacts of IMs with ER, inhibiting the departure of IMs from ER, thus resulting in the failed fusion of autophagosome with lysosome. Eventually, the autophagic flux is disrupted. This figure was modified from Figure 2 in Parzych and Klionsky (2014).

Autophagy is a cellular degradation and recycling mechanism that is highly conserved in all eukaryotes. There are three primary forms of autophagy in eukaryotic cells, namely, macroautophagy, microautophagy, and chaperone-mediated autophagy (CMA). Among them, macroautophagy is distinct from microautophagy and CMA in part because it relies on *de novo* formation of cytosolic IMs (autophagosome precursors), which sequester cargo and then elongate, close, and turn into mature autophagosomes and are finally transported by the fusion with lysosomes (Parzych and Klionsky, 2014) (Figure 1). The macroautophagy machinery has been widely investigated over the past decade. Nucleation of the IMs occurs at ER-associated autophagosome formation sites following induction by the Unc-51-like kinase 1 and 2 (ULK1/2) complex consisting of ULK1/2-ATG13-ATG101-FIP200. Elongation of the IMs is aided by the autophagy-related protein (ATG)12-ATG5-ATG16 like 1 (ATG16L1) complex; the class III phosphoinositide 3-kinase (PI3K) complex consists of ATG14-Beclin1-PIK3C3-PIK3R4 and LC3-II. The expanding membrane closes around its cargo and separates it from the ER to form a mature autophagosome. Eventually, the outer membrane of the autophagosome then fuses with the lysosomal membrane to form an autolysosome (Parzych and Klionsky, 2014). There has been substantial progress in understanding the molecular mechanisms underlying the regulation of this autophagic process, such as the cAMP-dependent protein kinase A and mammalian target of rapamycin (mTOR) complex 1 (MTORC1) pathways, which sense primarily carbon and nitrogen, respectively (Parzych and Klionsky, 2014). The presence of intracellular or extracellular protein aggregates resulting from the dysregulation of protein degradation pathways, such as those associated with the ubiquitin–proteasome pathway (UPP) or autophagy–lysosomal pathway (ALP), is associated with neurodegenerative disorders, including Parkinson’s disease (PD) (Shen et al., 2013), Alzheimer’s disease (AD), and amyotrophic lateral sclera (ALS) (English and Voeltz, 2013). The UPP and the ALP are the main systems responsible for intracellular protein degradation (Luo and Le, 2010). Soluble and short-lived proteins are mainly degraded through the UPP, while the clearance of insoluble protein aggregates is mainly mediated by the ALP.

The ER is a large, continuous, single-membrane-bound organelle found in eukaryotic cells, which forms dynamic contacts with other membrane-bound organelles, such as LDs (Li et al., 2019; Nguyen and Olzmann, 2019), mitochondria (Vance, 2014; Rodriguez-Arribas et al., 2017), Golgi (Salo et al., 2016), endosomes (Friedman et al., 2013; Dong et al., 2016), and IMs (Zhao et al., 2017) to maintain normal cellular processes. Communication between organelles at membrane contact sites plays important roles in cellular biological processes. The best-characterized inter-organelle connection involves mitochondria and a specialized domain found in the ER known as mitochondria-associated membrane (MAM) (Copeland and Dalton, 1959). MAM has been demonstrated to act as a signaling hub, playing a critical role in fundamental cell processes including mitochondrial function (Friedman et al., 2011; Szymanski et al., 2017), autophagy (Gelmetti et al., 2017; Gomez-Suaga et al., 2017), and apoptosis (Takeda et al., 2019; Yu et al., 2019), through the regulation of lipid metabolism and calcium (Ca^2+^) homeostasis (Szymanski et al., 2017; Romero-Garcia and Prado-Garcia, 2019). MAM dysfunction has been reported to be associated with various neurodegenerative diseases such as PD (Ottolini et al., 2013), AD (Area-Gomez et al., 2018), and ALS (Krols et al., 2016). MAM dysregulation contributes to accelerating neuronal death (Paillusson et al., 2016). However, it is still unclear whether MAM impairment is the cause or consequence in neurodegenerative diseases.

## Role of VMP1 in Autophagy

VMP1 was first identified as a protein responsive to pancreatitis-induced stress (Dusetti et al., 2002; Vaccaro et al., 2003). The *VMP1* gene codes for a protein of 406 amino acids (Dusetti et al., 2002). Structural analysis further revealed that VMP1 is a transmembrane protein containing six hydrophobic regions (Dusetti et al., 2002). Both the N- and C-termini of VMP1 are exposed to the cytosol (Tabara and Escalante, 2016; Zhao et al., 2017). The C-terminal region contains the putative ER retention signal, while the N-terminal region includes a conserved sequence with the potential to form an amphipathic alpha helix (Tabara and Escalante, 2016). VMP1 is localized to the ER and closely associated with, but clearly distinct from, markers for ER exit sites, the ER–Golgi intermediate compartment, and Golgi apparatus (Tabara and Escalante, 2016). VMP1 promotes the formation of intracytoplasmic vacuoles (Dusetti et al., 2002; Vaccaro et al., 2003).

Ropolo et al. (2007) firstly reported that VMP1 could trigger autophagy in mammalian cells. They showed that overexpression of VMP1 in mammalian cells could induce the formation of numerous vesicles that co-localize with LC3, a widely used autophagosomal marker. This autophagy-inducing activity can be blocked by the autophagy inhibitor 3-methyladenine (Ropolo et al., 2007). Overexpression of VMP1 induces autophagosome formation in cells even under sufficient nutrient and growth factor supply (Vaccaro et al., 2008). VMP1 is also found to interact with Beclin-1, a mammalian initiator of autophagy, and the VMP1 hydrophilic C-terminal region (ATG domain) is indicated to be essential for its interaction with Beclin-1 and induction of autophagy. Expression of VMP1 with a mutated ATG domain fails to induce LC3 recruitment and interaction with Beclin-1, which abolishes the triple co-localizations of VMP1, LC3, and Beclin-1 (Ropolo et al., 2007). However, Tabara and Escalante (2016) suggested that only a small proportion of VMP1 puncta is engaged in autophagy. They demonstrated that most autophagic structures co-localize with VMP1 puncta, while approximately only 5% of VMP1 puncta co-localize with autophagic markers. They also observed the close association of VMP1 with diverse organelles including mitochondria, peroxisomes, endosomes, and LDs. Moreover, not all VMP1 puncta appear to have contact with these organelles, and conversely, not all organelles have contact with VMP1. One VMP1 puncta can have contact with more than one organelle (Tabara and Escalante, 2016). Their studies indicate that the function of VMP1 is not limited in autophagic structures but remains diverse in connections with many organelles.

## Roles of VMP1 in ER–Membrane Contacts

### Role of VMP1 in ER–IM Contact

**FIGURE 2:**
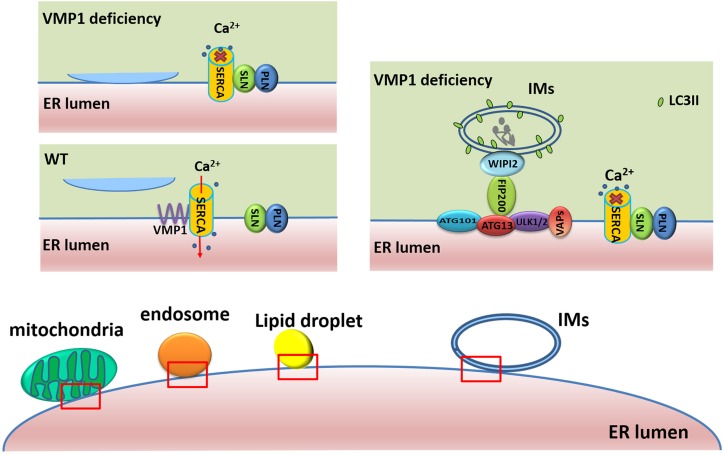
Roles of VMP1 in ER–membrane contacts. In WT cells, VMP1 competes with the inhibitory complex of PLN/SLN for interaction with SERCA to regulate the contacts of membrane structures (including mitochondria, endosome, and LDs) with ER. In VMP1-deficient cells, the interaction of SERCA with PLN/SLN leads to higher cytosolic Ca^2+^ levels and failure of the detachment of ER–membrane structure contacts. WIPI2 tethers IMs to the ER through interaction with ULK1/FIP200, and these interactions are reinforced by VMP1 depletion.

VMP1 has been shown to regulate ER–IM contacts (Figure 2), and its depletion extends the duration of omegasomes, ER-associated autophagosome formation sites enriched in phosphatidylinositol 3-phosphate (PI3P) (Zhao et al., 2018). During autophagosome biogenesis at omegasomes, punctate structures containing upstream ATGs, such as ULK1 and ATG14 tightly associate with the ER, while VMP1 transiently localizes to these punctate structures in a PI3K activity-independent manner (Itakura and Mizushima, 2010). However, loss of VMP1 activity leads to increased PI3P signaling in the ER (Calvo-Garrido et al., 2014), as well as increased contacts between IMs and the ER. Moreover, LC3 puncta also stably co-localize with double FYVE-containing protein 1 (DFCP1) and RB1 inducible coiled-coil 1 (RB1CC1, also known as FIP200) puncta, but these puncta are separable from those of lysosomal-associated membrane protein 1 (LAMP1)-labeled lysosomes. This demonstrates that depletion of VMP1 blocks autophagosome formation and leads to impaired autophagic flux after LC3 lipidation (Zhao et al., 2017). VMP1 is also reported to remain in IMs and subsequently in autophagic vesicle membranes (Ropolo et al., 2007). Furthermore, the tryptophan-aspartate repeat domain phosphoinositide interacting 2 (WIPI2) tethers IMs to the ER through interaction with ULK1/FIP200 and PI3P (Polson et al., 2010; Zhao et al., 2017), and these interactions are reinforced by VMP1 depletion via the modulation of sarcoplasmic/endoplasmic reticulum calcium ATPase (SERCA) activity (Zhao et al., 2017). They have indicated that SERCA/VMP1 reduces the cytosolic Ca^2+^ levels in the vicinity of IMs to trigger the detachment of ER–IMs contacts during autophagosome biogenesis. The SERCA activity is significantly lower in the microsome fraction from VMP1-depleted Cos7 cells than that from wild-type (WT) cells. They further demonstrated that VMP1 competes with the inhibitory complex of phospholamban (PLN)/sarcolipin (SLN) for interaction with SERCA. The interaction of SERCA2 with PLN and SLN is dramatically increased in VMP1 knockout (KO) cells compared with that in WT cells (Zhao et al., 2017).

Integral ER vesicle-associated membrane proteins (VAPs) such as VAPA and VAPB contribute to the establishment of contacts between the ER and various membranes by interacting with different tethering proteins (Hua et al., 2017; Johnson et al., 2018; Kirmiz et al., 2018; Zhao et al., 2018). VAPs and VMP1 are both demonstrated to modulate ER–IM contacts. While VAPs regulate ER–IM association, VMP1 regulates ER–IM dissociation. Depletion of VMP1 leads to an increased interaction between WIPI2 and ULK1/FIP200 (Zhao et al., 2017) and greatly promotes the interaction of VAPs with several autophagy-related proteins, including WIPI2 and ULK1/FIP200 (Zhao et al., 2018). The VAPB P56S mutation, which is linked with ALS in humans (Suzuki et al., 2009; Senturk et al., 2019), reduces ULK1/FIP200 interaction and impairs autophagy at an early step, similar to the effect observed in VAPA/B-depleted cells (Zhao et al., 2018). Dysfunction of any of these components can inhibit the formation and transfer of autophagosomes, thereby leading to impairment of the autophagic flux.

### Involvement of VMP1 in MAM

Mitochondria-associated membrane represents sites of contact between mitochondria and a specialized domain of the ER. MAM plays a crucial role in lipid metabolism and Ca^2+^ homeostasis, thereby regulating fundamental cellular processes such as changes in mitochondrial morphology, autophagy, and apoptosis (Montisano et al., 1982; Vance, 1990). In mammalian cells, MAM is a site for ATG accumulation and autophagosome formation (Hamasaki et al., 2013; Gelmetti et al., 2017). Recent studies have indicated that loss of VMP1 function results in a close association between the ER and mitochondria and an increase in ER–mitochondria contact sites (Tabara and Escalante, 2016; Zhao et al., 2017). Compared with that of WT cells, the average number of ER–mitochondria contacts is doubled in VMP1-depleted cells, and the proportion of mitochondrial membrane in contact with the ER is increased from 5.9 to 19% (Tabara and Escalante, 2016). Formation of ER–mitochondria tethering complexes, including VAPB/regulator of microtubule dynamics 3, mitofusin 1/mitofusin 2, and inositol 1,4,5-trisphosphate receptor type 1/voltage-dependent anion channel 1, is markedly increased in VMP1-depleted HeLa cells, and the levels of SERCA2 and other ATGs are also greatly increased in the MAM fraction of VMP1 KO HeLa cells when compared with control cells (Zhao et al., 2017). Maintenance of ER–mitochondria contacts is also modulated by VMP1 through inhibition of SERCA/PLN/SLN complex formation (Zhao et al., 2017). These results confirm the important roles of VMP1 in the communication between mitochondria and the ER. Depletion of VMP1 in both HeLa and Cos-7 cells leads to altered mitochondrial morphology (Tabara and Escalante, 2016), including altered size and the presence of inflated or absent cristae (Tabara and Escalante, 2016). These results suggest that VMP1 may contribute to the maintenance of mitochondrial morphology. Nevertheless, why mitochondria become spherical and swollen in VMP1-depleted cells requires further investigation.

### Participation of VMP1 in the ER–Golgi Network

The Golgi apparatus is the sorting station for processing, classifying, and packaging ER-derived proteins and plays an important role in the synthesis and transport of proteins destined for secretion. Numerous molecules contribute to ER–Golgi production and processing networks, the components of which are vital for the maintenance of normal cellular function (McCaughey and Stephens, 2019; Venditti et al., 2020). Dysregulation of VMP1 in the social amoeba *Dictyostelium discoideum* is recently shown to result in a severe phenotype that compromises its growth and development; however, this phenotype can be rescued by the expression of mammalian VMP1 in VMP1-null *Dictyostelium* cells, suggesting a functional conservation of the protein among different species (Calvo-Garrido et al., 2008). VMP1 is necessary for ER integrity in *Dictyostelium*. Mutant cells exhibit a fragmented ER and disorganized Golgi apparatus (Calvo-Garrido et al., 2008), similar to the whorl structures observed in the ER of VMP1-depleted Cos-7 cells (Tabara and Escalante, 2016). Rapid accumulation of autocrine proliferation repressor protein A (AprA), a protein secreted through the ER–Golgi transit pathway, is observed in the extracellular media derived from *Dictyostelium* cells expressing control VMP1. However, AprA is not observed in the media obtained from VMP1 mutant cells, suggesting that VMP1 may also be involved in protein secretion. Additionally, endocytosis and exocytosis are also severely impaired in cells expressing mutant VMP1 (Calvo-Garrido et al., 2008).

### Association of VMP1 in ER–LD Contact

Lipid droplets are membrane-bound organelles consisting of a core of neutral lipid surrounded by a phospholipid monolayer (Gao et al., 2019). LDs store excess lipids and play important roles in lipid metabolism during numerous cellular processes. Several studies have highlighted the importance of LDs in the nervous system (Liu et al., 2015, 2017). Glial-derived LDs and neuron-to-glia lipid transfer are important for preventing neuronal lipotoxicity (Pennetta and Welte, 2018). The ER is closely related to LD formation. LD biogenesis begins in the ER bilayer, after which the LDs bud off into the cytoplasm (Olzmann and Carvalho, 2019). After their initial formation, LDs retain a functional and morphological connection with the ER, as indicated by their dynamic cargo exchange and the close distance between these two organelles (Walther et al., 2017). It is currently unknown which molecules are responsible for the maintenance of ER–LD contacts. However, VMP1 is recently shown to be an important molecule for ER–LD contacts, as well as LD morphology (Zhao et al., 2017). VMP1-depleted HeLa cells, VMP1-depleted Cos-7 cells, and VMP1 mutant worms display enlarged LDs, although the patterns differ between HeLa and Cos-7 cells (Tabara and Escalante, 2016; Zhao et al., 2017). In VMP1-depleted HeLa cells, the LDs are larger, and there is an accumulation of LD clusters, while in VMP1-depleted Cos-7 cells, the accumulation of LD clusters is not significant (Tabara and Escalante, 2016). Contacts between the ER with LDs are greatly increased in *shVMP1* HeLa cells (Zhao et al., 2017). In control cells, the percentage of ER–LD contact sites increases from 31.5 to 72.7%, whereas the average percentage of the total perimeter of LDs that are close to the ER is elevated from 2.2 to 16.3% in VMP1-depleted cells. Electron tomography images also indicate that loss of VMP1 enhances the association between the ER and LDs (Zhao et al., 2017). Contacts between these two organelles are also regulated by VMP1 through inhibition of SERCA/PLN/SLN complex formation, which is further evidenced by the increase in the number of ER–LDs contact sites induced by the SERCA-specific inhibitor thapsigargin (Zhao et al., 2017).

### Role of VMP1 in ER–Endosome/Endolysosome Contact

During the past two decades, several functions for ER–endosome/endolysosome contact sites have been revealed, including lipid trafficking, cargo sorting, endosome trafficking, and fission (Raiborg et al., 2015a, b). The ER has been shown to make contacts with endosomes shortly after they are formed, and the degree of ER–endosome contact increases as early endosomes mature into late endosomes (Friedman et al., 2013).

Recently, VMP1 puncta are found to be closely associated with Rab5- and Rab7-marked endosomes, with Rab7-containing late endosomes showing the most frequent co-localization with VMP1 (Tabara and Escalante, 2016). VMP1 depletion leads to a marked increase in the number of contacts between the ER and endosomes/endolysosomes, as evidenced by the greater percentage of endosomes/endolysosomes in contact with the ER, as well as the greater contact length and increased formation of the VAPA/oxysterol binding protein like 1 (ORP1L) complex (Zhao et al., 2017). Moreover, the PI3K inhibitor wortmannin reduces the abundance of contacts between the ER and endosomes/endolysosomes, indicating that PI3P contributes to the enhanced ER contacts observed in VMP1-depleted cells (Zhao et al., 2017). The contacts between the ER and endosomes/endolysosomes are also reportedly regulated by interaction between VMP1 and SERCA (Zhao et al., 2017).

## Potential Implications of VMP1 in Neurodegenerative Disorders

**FIGURE 3:**
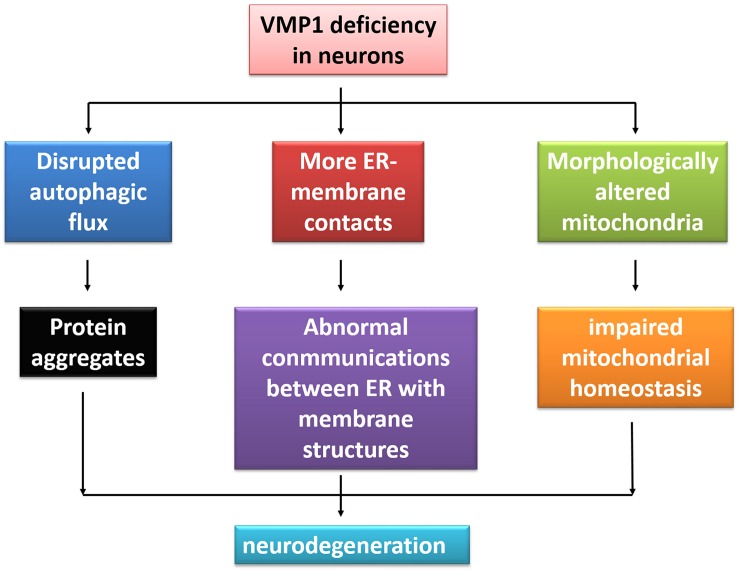
The illustration of VMP1 deficiency in neurodegeneration. Deficiency of VMP1 results in more ER–membrane contacts, disrupted autophagic flux, and morphologically altered mitochondria, bringing the abnormal communications between ER with membrane contacts within cells, protein aggregates, and impaired mitochondrial homeostasis. Finally, this may lead to neurodegeneration.

VMP1 is initially linked with pancreatitis (Dusetti et al., 2002). Over the past decade, other disorders apart from pancreatitis have been reported to be associated with VMP1, such as cancers and inflammatory bowel disease (Ventham et al., 2016; Amirfallah et al., 2019). High expression of VMP1 is a potential marker in human epidermal growth factor receptor 2-positive breast cancer (Amirfallah et al., 2019). Since the major roles of VMP1 are associated with ALP (Ropolo et al., 2007) and the dysfunction of the system may contribute to various neurodegeneration conditions (Menzies et al., 2017; Miki et al., 2018), it is suspected that VMP1 may have an implication in neurodegenerative disorders (Figure 3).

Autophagy is the major degradation pathway involved in the clearance of protein aggregates, the presence of which is closely associated with several neurodegenerative diseases (Hara et al., 2006). Activating autophagy through cAMP-response element binding protein-regulated transcription coactivator (Crtc, also known as MTORC1) inhibition increases the degradation rate of the huntingtin protein (HTT) and ameliorates the neurodegeneration-like effects generated in mouse and fly models of Huntington’s disease (Ravikumar et al., 2004). Using a novel *Caenorhabditis elegans* model to investigate the role of autophagy in ALS, English and Voeltz (2013) found that increased autophagy could protect *C. elegans* motor neurons against the toxicity associated with aggregation of mutant superoxide dismutase 1, which causes age-dependent motor defects. Therefore, activated autophagy might also exert beneficial effects against other neurodegenerative diseases. Overexpression of VMP1 induces autophagosome formation in mammalian cells even under sufficient nutrient and growth factor supply, which might lead to clearance of accumulated proteins involved in neurodegenerative disorders (Vaccaro et al., 2008).

The autophagy pathway is complex, with multiple steps and modes of regulation. This makes identifying potentially minor perturbations in the pathway difficult. Therefore, in some cases, the findings for autophagy involved in neurodegenerative disease pathogenesis appear controversial (Menzies et al., 2017). However, multiple nervous system-specific KO mouse models have been generated to allow analyses of the roles of autophagy in neuronal function. Conditionally, *ATG5* gene KO in mouse neurons reduces autophagy in the baseline level, resulting in the accumulation of ubiquitinated proteins and p62 and ultimately leading to neurodegeneration (Hara et al., 2006). Conditionally knocking out *FIP200* and *ULK1/2* in mouse neurons, respectively, leads to a reduced survival rate and early-onset, progressive neurodegeneration (Liang et al., 2010; Joo et al., 2016). In VMP1 mutant *Dictyostelium* cells, there is extensive accumulation of very large ubiquitin-positive protein aggregates containing LC3 and the putative *Dictyostelium* p62 homolog, which are also observed in several human neurodegenerative disorders, including AD and PD (Calvo-Garrido and Escalante, 2010). VMP1-null cells of the green alga *Chlamydomonas reinhardtii* exhibit defective cytokinesis, aberrant cell shapes, enlarged mitochondria, accumulation of triacylglycerides, and enlarged starch granules due to the presence of defective autophagosomes (Tenenboim et al., 2014).

PD is characterized by pathophysiologic loss or degeneration of dopaminergic neurons in the substantia nigra of the midbrain and, typically, by the presence of α-synuclein (α-syn) inclusions. These inclusions predominantly localize to axons even in the early stages of the disease (Volpicelli-Daley et al., 2014). In primary neuron cultures with α-syn inclusions, although lysosomal function appears normal, maturation of autophagosome and its fusion with the lysosomes are decreased, resulting in a decrease in protein degradation (Volpicelli-Daley et al., 2014). It is interesting to note that this alteration in autophagosome trafficking seems to be consistent with that observed in VMP1-depleted cells, because depletion of VMP1 blocks autophagosome maturation as well as dissociation with ER, leading to the failed fusion with lysosome (Ropolo et al., 2007; Zhao et al., 2017). It is hypothesized that the VMP1 deficiency in neurons may induce the pathology of α-syn inclusions accumulating at axons.

There are other diseases whose pathogenesis is related to the damage of the autophagy lysosomal system, and VMP1 may play roles in regulating the development of these diseases. Polysulfatase deficiency (MSD) is a rare but more damaging disease in which patients develop complex multisystem phenotypes due to impaired sulfatase activity. There is a barrier to the fusion of vesicles and lysosomes. A large accumulation of autophagic substrates such as polyubiquitinated proteins and dysfunctional mitochondria indicate autophagic dysfunction in MSD mice (Fecarotta et al., 2018). Glucocerebrosidase V394L mutant homozygous mice and saposin C mutant mice are often used as animal models of Gaucher disease, which show punctate p62 aggregates in their neurons and astrocytes, while undigested materials are found in the axonal vesicles with accumulation of autophagic substrates (Aflaki et al., 2017).

Mitochondrial damage is one of the main causes of neurodegenerative diseases, including AD, PD, and ALS. In brief, accumulated damage to mitochondria may collaborate with soluble β-amyloid and phosphorylated Tau to induce synapse loss and cognitive impairment in AD (Jeong, 2017). Damaged mitochondria, oxidative damage of multiple proteins, and increased oxidative stress in the substantia nigra–striatum pathway were observed in substantia nigra samples from PD patients (Ikawa et al., 2011). Several PD-associated proteins are also closely related to mitochondrial function, like α-syn, Parkin, PTEN-induced kinase 1, and DJ-1, and mutations of these proteins can lead to damage of the mitochondrial structure, function, and transfer (Pickrell and Youle, 2015; Rocha et al., 2018). ALS is an incurable neurodegenerative disease which is pathogenically based on the mitochondrial alteration of motor neurons, causing progressive neuron death (Lin and Beal, 2006). VMP1-null cells display an increased abundance of spherical and swollen mitochondria (Zhao et al., 2017). In neurons, damaged mitochondria are efficiently cleared through mitophagy, a kind of selective macroautophagy process that plays a fundamental role in mitochondrial homeostasis, energy supply, and neuronal survival (Lou et al., 2019). However, mitophagy is also disrupted in VMP1 KO cells. Impaired mitophagy and accumulation of damaged mitochondria are present in VMP1-null cells (Tabara and Escalante, 2016; Zhao et al., 2017), which may induce or aggravate neurodegeneration.

VMP1-null cells also show a close association between the ER and mitochondria, more ER–mitochondria contacts, and increased levels of tethering proteins in MAM sites, which disrupt normal communications at MAM (Zhao et al., 2017). In both familial and sporadic AD, there is an increased interaction between the ER and mitochondria, as well as in MAM function (Area-Gomez et al., 2012). Several neurodegeneration-related proteins, including Parkin, HTT, and presenilin-1, are localized to MAM, and dysregulation of many of these proteins is implicated in neurodegenerative diseases. Although relatively few studies have analyzed the direct relationship between VMP1 and these neurodegeneration-related proteins, current evidence strongly indicates that VMP1 has a potential role in neurodegenerative diseases through its role as a critical regulator of ER–membrane contacts and autophagy.

Numerous ER–endosome/endolysosomal tether proteins are associated with neurodegeneration. VAPs, which are associated with familial ALS in humans, control ER–endosome/endolysosomal contacts and endosomal migration via ORP1L (Rocha et al., 2009). However, VMP1 depletion results in larger contact length and increased VAP/ORP1L complex formation (Zhao et al., 2017). This interference may break the balance of ER–endosome/endolysosomal communications.

## Conclusion

Increasing evidences indicates that VMP1 plays important roles in autophagy by regulating the connections between the ER and IMs. Depletion of VMP1 results in a greater number of contacts between the ER and IMs, mitochondria, LDs, and endosomes/endolysosomes. VMP1 acts as a regulator of the extent and frequency of ER contacts with these organelles, rather than as a tether. Communication between membrane structures at contact sites, such as content exchange and signaling transduction, is important for the maintenance of normal cellular activities. However, the membrane contact sites are always highly dynamic. Although contact sites may have diverse roles, VMP1 functions as a structural dissociator at these contact sites. The dynamicity of the contacts can be inhibited by VMP1 depletion, and spherical and swollen mitochondria are observed in VMP1-depleted cells. In *Dictyostelium* cells, a VMP1-related gene has pleiotropic functions in the secretory pathway, organelle biogenesis, and the onset of multicellular growth and development. Moreover, VMP1 mutations can induce ubiquitin-positive protein aggregates and extensive accumulation of the *Dictyostelium* p62 homolog. Abnormal protein accumulation is a feature of neurodegenerative disorders such as PD and AD. VMP1 is critical in the early stage of autophagosome formation, and overexpression of VMP1 can trigger autophagy, which could lead to neuronal clearance of accumulated proteins that is the hallmark of many neurodegenerative disorders.

## Author Contributions

PW and DK wrote the manuscript. WL designed the concept and manuscript outline and revised the final version. All authors agreed to be accountable for the content of the work.

## Conflict of Interest

The authors declare that the research was conducted in the absence of any commercial or financial relationships that could be construed as a potential conflict of interest.
